# J Young Pharm. 2011: What are we aiming at?

**DOI:** 10.4103/0975-1483.76412

**Published:** 2011

**Authors:** Ahmed KK Mueen

**Affiliations:** *Editor-in-Chief, J Young Pharm. mueen.ahmed@gmail.com*

## QUALITY PUBLICATION AND ISI INDEXING

A journal is always judged based on the quality of papers, such as original papers, reviews, editorials, letters to the editor, being published and these articles play a most crucial role in research process. A high-quality publication is a means of science communication and collaboration of scientists in their field of expertise.[1] High-quality publications attract more attention of other researchers, stimulating new ideas and becoming highly cited. They boost authors’ research profile, strengthen positions of institutions supporting scientific productivity, and increase chances to get funding for future research studies. Currently, in most countries, academic promotion of an individual, recruitment, and distribution of financial sources for research are all subjected to the evaluation of a researcher/institution profile in world renowned indexing systems and catalogues, such as PubMed, Scopus, Index Copernicus, Institute for Scientific Information (ISI), etc.[2]

**Figure 1:**
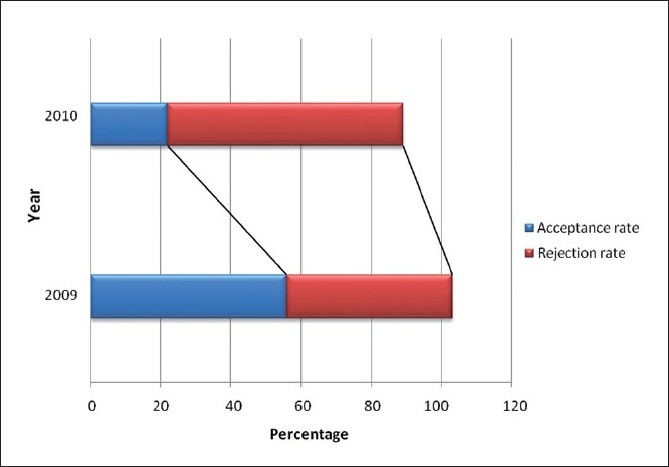
Acceptance and rejection rate of *J Young Pharm*. during the past 2 years (Courtesy: journalonweb)

To achieve leading positions in academics and scientific field, academic and research institutions worldwide are now strongly encouraged to improve the quality of research studies and to publish papers in peer-reviewed journals visible in most prestigious indexing databases.[3] *J Young Pharm*. is now indexed with Caspur, Chemical Abstracts, DOAJ, EBSCO Publishing’s Electronic Databases, Expanded Academic ASAP, Genamics JournalSeek, Global Health, Google Scholar, Health and Wellness Research Center, Health Reference Center Academic, Hinari, Index Copernicus, OpenJGate, PrimoCentral, ProQuest, PubMed, Pubmed Central, SCOLOAR, SCOPUS, SIIC databases, Summon by Serial Solutions, and Ulrich’s International Periodical Directory. We are aiming at inclusion in Institution of Scientific Information (ISI) during this year. *J Young Pharm*. is also expecting SJR Ranking by the end of this year as we are already in SCOPUS.Figure 1 and Table 1 show the rate of acceptance/rejection rate for the past 2 years; we have drastically improved the refining of papers in the last year, as it can be seen, our rejection rate is higher when compared to the year 2009. Combined efforts of authors, reviewers, editorial board members, and of course, publisher will further improve the quality of papers being submitted and published.

**Table 1:** Number of articles submitted along with accepted/rejected ratio during the year 2010

Article type	Submitted	Accepted	Rejected	Under review
Editorial	16	5 (31)	10 (63)	1 (6)
General Articles	48	8 (17)	34 (71)	6 (13)
Letter to Editor	5	2 (40)	1 (20)	2 (40)
Original Article	155	30 (19)	102 (66)	23 (15)
Pharm Analysis	6	1 (17)	5 (83)	0 (0%)
Pharm Chemistry	6	5 (83)	2 (33)	-1 (-17)
Pharm Management	1	1 (100)	0 (0)	0 (0)
Pharm Marketing	1	0 (0)	1 (100)	0 (0)
Pharmaceutics	4	1 (25)	3 (75)	0 (0)
Pharmacognosy	11	2 (18)	9 (82)	0 (0)
Pharmacology	10	3 (30)	8 (80)	-1 (-10)
Review Article	1	0 (0)	1 (100)	0 (0)
Total decisions	234	58 (25)	176 (75)	
Total articles	264	58 (22)	176 (67)	30 (11)

Figures in parentheses are in percentage, Courtesy: journalonweb

## INCLUSION OF DOI IN THE CITED REFERENCES

Many of the articles submitted to JYP have been observed for the inaccurate and incomplete references, failing to locate the source for further information about the submitted manuscript. It makes our reviewers more concerned on correctness of these references. Uniform reference locators (URLs) or digital object identifiers (DOIs) are very useful in locating references on the Web, and authors are requested to provide the link/DOI in their reference list, which will further smoothen the peer-review process. Hence, JYP submission in the year 2011 will ask for DOI (if available) or full text link for the all the references cited in the submitted manuscripts.

## INCLUSION OF MORE REVIEWERS

**Figure 2:**
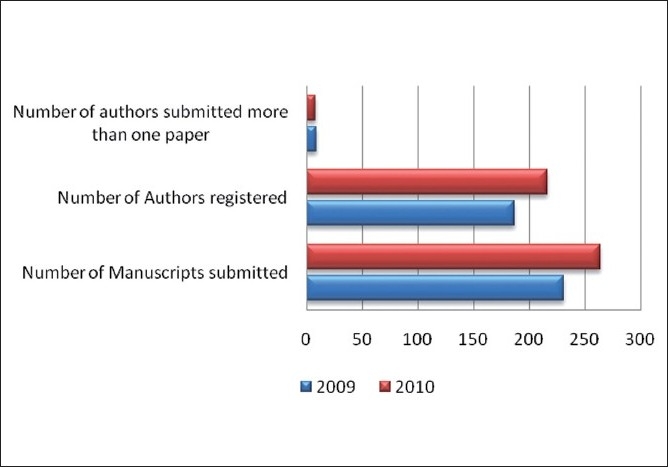
Manuscript submissions to *J Young Pharm*. during the past 2 years (Courtesy: journalonweb)

On behalf of the editorial board members, I would like to thank all the reviewers who have helped us in refining the papers submitted to *J Young Pharm*. We acknowledge them fully for their contribution and we have published a list of all of our reviewers in page 73. We are presenting a brief report on the performance of *J Young Pharm* during the year 2010 [Figure 2]. Performance of our reviewers is included in Table 2 and summary of JYP results for the year 2010 is presented in Table 3. We are constantly adding new reviewers; hopefully, we will have a large number of active and dedicated reviewers by this year end. Our reviewers are on time in reviewing the papers for us. Not all the time, our reviewers’ response toward my invitation is fruitful. Most of our reviewers remain busy with their busy research or academic schedules. We are extremely thankful to them for spending their precious time for refining articles of *J Young Pharm*.

**Table 2:** The time scale submitted to first decision for the year 2010

Number of articles submitted	264
Days to suggest reviewers	5.30 (0, 42)
Days taken by reviewers	14.91 (0, 47)
Days taken by Editor for decision	9.85 (0, 87)
Days until paper is under review	30.06
Days from first decision until revision arrives	11.55 (0, 74)
Days for Editor to take decision	3.60 (0, 11)
Articles re-reviewed	7
Days for re-review by reviewers	6.40 (0, 15)
Days to send revision decision by Editors	3.60 (0, 11)
Days from revision receipt to revision decision	13.6
Days from first submission to acceptance	121.96 (1, 186)
Days from acceptance to publication	60.12 (12, 134)

Courtesy: journalonweb

**Table 3:** *JYP* results for the year 2010

Total number of authors registered with the site	624
Number of authors who have submitted manuscripts	216
Number of authors who have submitted more than one	8
manuscripts	
Number of manuscripts from abroad	46(17%)
Number of original articles from abroad	27 (17%)

Courtesy: journalonweb

## EXPANSION AND RESTRUCTURING OF EDITORIAL BOARD

Members of our board take a posture of being supportive and nurturing to authors who submit papers to the journal. Instead of making our mark by citing how many papers we reject, we want to be known for helping authors become better writers and researchers. To this end, our reviewers work with the editorial leadership team as part of an editorial community which unconditionally accepts all authors who submit their papers to the journal. In turn, we ask these authors to participate wholeheartedly in the editorial process of writing and rewriting. In this approach, reviewers and editors become mentors to the authors, and as a team, we work collaboratively to produce the best papers and journal possible.

An editorial board member will be expected to perform the following responsibilities:

- review two to three new papers per quarter;
- review the revised manuscripts;
- produce supportive and useful reviews;
- participate in internal quality improvement activities (e.g., answer surveys, review new forms and procedures, provide feedback); and
- communicate with editors in a timely and professional manner.
- Benefits of the position include the following.
- Joining an exciting virtual community dedicated to improving qualitative research scholarship and scholars.
- Collaborating with an international editorial leadership team.
- Mentoring authors from around the world.
- Learning a novel and effective approach to reviewing scholarly papers.
- Receiving editorial reviewing resource materials.
- Participating in research projects to improve the editorial process.
- Learning to use new software applications for reviewing and editing.
- Improving your own scholarly writing.
- Acquiring knowledge on the latest innovations in qualitative inquiry.
- Gaining a valuable credential for tenure and promotion review.

*J Young Pharm*. will be expanding its editorial board during the year 2011. Active reviewers will be now included as editorial board members. Interested authors can write to us along with their brief profile for possible inclusion.

## FINANCIAL STRENGTHENING

*J Young Pharm*. is an official publication of InPharm Association and is being managed by self-funding. Our site (http://www.jyoungpharm.in) is fully dynamic, and the papers being published are now available in multiple formats such as PDF, Full text, ePUB, Sword repository plugin, eBook, etc.[4] The present global requirement of journals in different output formats is a costly process. We request all the colleges and authors to subscribe the journals and further strengthen us financially. There will be nominal pre-press charges for all the manuscripts getting published from the year 2011. This policy will remain throughout the year until next notification.

## MOST CITED PAPERS

I would like to list all the most cited papers from the year 2009.[5–10] I encourage the authors to cite *J Young Pharm*. papers and utilize them in their research writings. Our citations are available in multiple formats from our website (http://jyoungpharm.in/currentissue.asp?sabs=n) and Citeulike page (http://www.citeulike.org/user/jyoungpharm) as well.
